# Application of Polymers in Advanced Materials for Enamel and Dentine Remineralization

**DOI:** 10.1021/acsbiomaterials.5c02188

**Published:** 2026-04-13

**Authors:** Zichen Wu, Le Zhang, Jingwen Wu, Yanchuan Guo, Quanli Li, Hai Ming Wong

**Affiliations:** † Paediatric Dentistry and Orthodontics, Faculty of Dentistry, 71025 The University of Hong Kong, 34 Hospital Road, Hong Kong SAR 999077, PR China; ‡ Chinese Academy of Sciences, 74703 Technical Institute of Physics and Chemistry, Beijing 100190, PR China; § School of Future Technology, University of Chinese Academy of Sciences, Beijing 100049, PR China; ∥ Department of Stomatology, Longgang Otorhinolaryngology Hospital, Institute of Oral Science, No. 186 Huangge Road, Shenzhen 518172, PR China; ⊥ Key Lab of Oral Diseases Research of Anhui Province, College and Hospital of Stomatology, 12485 Anhui Medical University, Meishan Road, Hefei 230032, PR China

**Keywords:** enamel remineralization, dentine remineralization, biomineralization, biomimetic mineralization, polymer

## Abstract

Dental caries, the most common oral disease with a worldwide burden, causes structural damage to enamel and dentine. Considering the limitations of conventional restorative approaches and the dynamic nature of caries, biomimetic remineralization strategies offer a promising alternative for restoring tooth structure. Central to many of these strategies is the use of polymers to emulate the role of macromolecular templates in natural mineralization processes. This review summarizes the polymers applied for biomimetic mineralization of enamel and dentine according to their primary functions. Furthermore, perspectives on the trends and challenges for future research on polymeric biomaterials for enamel and dentine repair are also presented.

## Graphical abstract

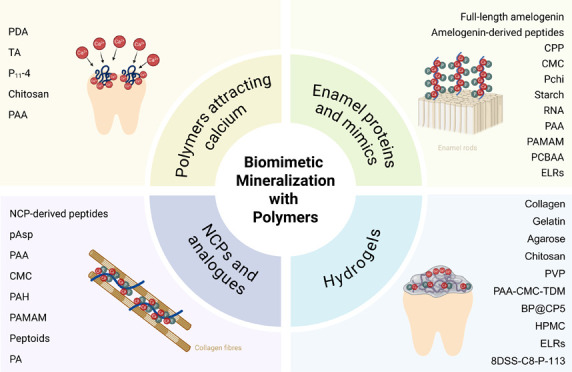

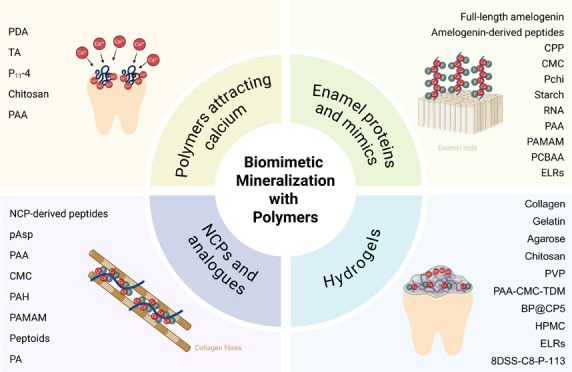

## 1 Introduction

Enamel and dentine are two fundamental components of dental hard tissues, with enamel mainly protecting inner structures of the tooth and dentine mainly supporting the enamel, forming the bulk of the tooth and protecting the pulp. However, dental caries, one of the most common oral diseases, damages or even destroys the structures of enamel and dentine, impairing the normal functions of teeth and imposing significant socioeconomic burdens, affecting approximately 2.5 billion people worldwide.^1^ Typically, caries begin at the enamel surface, where the crystalline structure undergoes demineralization due to acids produced by bacteria within cariogenic biofilms. At the early stage of the disease, the decay can be arrested or reversed if the protective factors overpower the pathological factors and shift the balance to remineralization.^2^ If the situation is not controlled, the progression of the disease would increase mineral loss leading to microcavitations or lesions in enamel or, more severely, cavitations in dentine and eventually pulpitis.

Nowadays, the most common technique to treat dental caries and restore the tooth structure is restoration with filling materials after the removal of decayed tissues. Despite the widespread clinical application, those dental fillings often exhibit compromised interfacial bonding with native tooth tissues, resulting in restoration detachment or secondary caries, and require further treatment.^3^ Therefore, remineralization, as a potential alternative strategy, has been extensively studied, which not only obviates the need for synthetic fillings but also etiologically intervenes in cariogenic processes. Fluoride agents and calcium phosphate supersaturated solutions are commonly used for the clinical remineralization of enamel; however, these agents have limited remineralization efficiency and fail to reproduce the original microstructure. Thus, the development of novel approaches to promote remineralization efficiency and recovering the microstructure is highly warranted in caries treatment.

Enamel as the hardest tissue with a highly mineralized structure in the human body consists of 96 wt % hydroxyapatite (HA), 1 wt % organic matrix, and 3 wt % water, while dentine is less mineralized than enamel due to a higher proportion (20 wt %) of organic matrix mainly composed of type I collagen and less proportion (70 wt %) of minerals.^4,5^ Both enamel and dentine possess complex hierarchical structures that originate from the natural process of biomineralization. Biomineralization is an intricate biological process involving the controlled nucleation and growth of inorganic mineral crystals, primarily hydroxyapatite, within an organic matrix. This process is tightly regulated by a variety of organic molecules, which range from small organic compounds, such as ions and small peptides, to larger macromolecules, including proteins, peptides, and nucleic acids. These organic components serve as templates, guides, and regulators that influence crystal size, shape, orientation, and overall organization, resulting in the highly organized and functional microstructures seen in mineralized tissues like enamel and dentine.^6^

Inspired by the natural mechanisms of biomineralization, researchers have sought to emulate these processes by exploring various polymers and organic molecules as artificial templates.^7^ The goal is to structurally and functionally mimic the role of natural organic matrices in guiding mineral deposition. By incorporating specific polymers that can interact with inorganic minerals, scientists aim to control nucleation and growth processes more precisely, ultimately replicating the hierarchical architecture of native enamel and dentine.^8^ These biomimetic strategies involve designing polymeric templates that can facilitate mineralization in a manner similar to that of natural processes, promoting the formation of organized mineral structures. Such approaches aim to enhance remineralization efficiency—restoring mineral content to demineralized tissues—and, in some cases, achieve regeneration of the microstructures of enamel and dentine.^9^ Successful implementation of these techniques holds promise for advanced dental therapies, enabling the repair of damaged or decayed dental tissues in a manner that restores their natural function, durability, and structural integrity.

This review adopts a comprehensive functional classification framework that categorizes polymeric systems based on their specific roles and mechanisms in biomimetic remineralization. Additionally, it provides a more in-depth mechanistic focus, elucidating how different polymers influence mineral nucleation, growth, and organization. Furthermore, this review emphasizes emerging trends and the development of multifunctional polymeric systems that combine remineralization with additional biological or therapeutic functionalities. These aspects collectively distinguish this review from prior literature and provide a more integrated perspective on recent advancements in organic polymers involved in enamel and dentine repair.^10–13^ The polymers are categorized into four types based on their mechanisms for promoting mineralization: (1) simply attracting binding calcium ions and providing inorganic sources for mineralization, (2) mimicking the enamel proteins, (3) mimicking the noncollagenous proteins, and (4) providing mineralization-favorable environment (mainly in the form of hydrogels). The specific polymers, their mechanisms of action, key functional groups, and the main findings of mineralization studies on enamel and dentine are summarized in Table 1.

**Table 1:** Summary of Polymers Applied in Enamel and Dentine Remineralization

polymer	key moieties	main mechanisms for remineralization	results	refs
PDA	catecholamine	chelates Ca^2+^	in vitro: complete occlusion of dentinal tubules	^17,18^
TA (with SAP)	pyrogallol	chelates Ca^2+^	in vitro: regeneration of uniform nanorod-like HAP on enamel with great surface microhardness recovery and strong adhesion force	^19^
			in vivo: remineralization of HAP in the oral cavity of rats	
P_11_-4	glutamine residues	binds Ca^2+^	clinical trials have shown superior remineralization efficacy compared to pure application of fluoride	^22^
chitosan	amino and hydroxyl groups	binds Ca^2+^; providing mineralization-favorable environment	in vitro: enhanced WSL remineralization with BAG; organized crystallization, antimicrobial property, pH responsiveness, and adhesiveness when loaded with amelogenin and QP5; superior remineralization effects to NaF varnish when loaded with nHA	^23,125–131^
PAA	carboxyl groups	binds Ca^2+^; mimics amelogenin and NCPs	in vitro: enhanced WSL remineralization with BAG; comparable levels of WSL remineralization to CPP-ACP; promoting intrafibrillar mineralization	^26,65,92–96^
rP172	multiple domains as amelogenin	controls crystal nucleation and oriented growth; stabilizes ACP	in vitro: formation of oriented FHA needles; enhanced crystallinity	^33^
LRAP; TRAP; QP5	derived amelogenin sequences (Gln–Pro–X, N-terminus, C-terminus)	mimics amelogenin	in vitro: oriented enamel-like HA growth	^37–39,41,42,44–46^
			in vivo: mineral gain and reduced caries progression in a *S. mutans*-infected rat model	
PTL/C-AMG	C-terminus; phase-transited lysozyme (mimic N-terminus)	mimics amelogenin	HA layer harder than native enamel; strong bonding to substrate	^47^
CPPs	acidic residues particularly the –S (p) S (p) S (p) EE-	mimics amelogenin	evidence-based research confirms the effectiveness of CPP-ACP and CPP-ACFP for caries management	^53^
CMC	carboxyl and amino groups	mimics amelogenin and NCPs	in vitro: enamel-like crystal regrowth; scaffold for dentine repair; deposition of HA within fibrils	^56–58,97^
Pchi	phosphate groups	mimics amelogenin	in vitro: comparable remineralization effect on enamel lesions to fluoride	^59^
starch	hydroxyl groups	mimics amelogenin	in vitro: on-site mineralization of both enamel and dentine	^62^
RNA	phosphate groups	mimics amelogenin	in vitro: formation of dense, organics-free enamel-like apatite	^63^
			in vivo: epitaxial growth of dense and oriented crystals on enamel in the oral cavity of rats	
PAMAM dendrimers (COOH/NH_2_/OH)	carboxyl, amino, and hydroxyl groups	mimics amelogenin and NCPs	in vitro: rapid formation of HA crystals like intact enamel; inhibited *S. mutans* adhesion; intrafibrillar mineralization of dentine and dentinal tubule occlusion	^67–69,71,102–108^
PCBAA	quaternary ammonium and carboxyl groups	mimics amelogenin	in vitro: promoted rapid remineralization for enamel and dentinal tubule occlusion	^73^
			in vivo: formation of dense mineral layer resistant to biting forces and completely occluded dentinal tubules with antibiofilm property in a rat model	
ELRs	-	mimics amelogenin; providing mineralization-favorable environment	in vitro: recreate the microstructure of the different anatomical regions of enamel with epitaxial growth of apatite nanocrystals; an average of 78 μm mineralization depth after 14 day mineralization	^76,142^
8DSS	aspartate–serine–serine (DSS)	mimics NCPs	in vitro: mineral deposition on both demineralized dentine surface and dentinal tubules, with improved mechanical properties and dentinal occlusion	^77–80^
peptides derived from DMP-1	C-terminus	mimics NCPs	in vitro: promoted intrafibrillar mineralization of dentine collagen, and the remineralization of enamel lesions	^83–85^
pAsp	carboxyl groups	mimics NCPs	in vitro: promoted intrafibrillar mineralization; restores nanomechanical properties of dentine; hierarchical HA growth	^88–91^
PAH	primary amine groups	mimics NCPs	in vitro: promoted dentine occlusion and intrafibrillar mineralization	^99–101^
peptoids	alternating hydrophobic and hydrophilic groups	mimics NCPs	in vitro: enhanced peritubular dentine mineralization and mechanical recovery	^111^
PA	catechol and hydroxyl groups	mimics NCPs	in vitro: accelerated intrafibrillar mineralization; hierarchical mineral organization and mechanical properties comparable to natural dentine	^114^
collagen hydrogel (with ODAM and AMTN)	-	providing mineralization-favorable environment	in vitro: enamel- or dentine-like crystal formation; biochemical compatibility	^121,122^
PVP/ACP	-	providing mineralization-favorable environment	in vitro: formation of continuous FHA layer; dentinal tubular occlusion	^132^
gelatin	amino groups	providing mineralization-favorable environment	in vitro: epitaxial growth of FAP crystals parallel to the organic matrix	^135^
agarose	-	providing mineralization-favorable environment	in vitro: formation of enamel prism-like tissue and deep occlusion of dentinal tubules when loaded with CaCl2	^119,136–138^
			in vivo: highly organized HA crystals on dentin surface and occlusion of dentinal tubules in a rabbit model	
HPMC	hydroxyl and methoxyl groups	providing mineralization-favorable environment	in vitro: early mineralization of demineralized dentin after 24 h and increasing mineralization of the whole demineralized dentin (3–4 μm) after 72–96 h	^140^
PAA-CMC-TDM	carboxyl groups	providing mineralization-favorable environment	in vitro: maintenance of the bioactivity of TDM and induce osteogenic and odontogenic differentiation of DPCs	^143^
			in vivo: promotion of the in situ regeneration of defective tooth and bone tissues	
BP@CP5	catechol groups (from catechol-modified chitosan)	providing mineralization-favorable environment	in vitro: highly efficient bactericidal and remineralization-promoting effects	^144^
			in vivo: prevention of caries formation in a rat model	
8DSS-C8-P-113	Asp–Ser–Ser repeats	providing mineralization-favorable environment	in vitro: in situ remineralization of enamel with generation of a dense and oriented layer of HA crystals and substantial recovery of microhardness; *S. mutans*-specific antibiofilm activity	^145^
			in vivo: effective reduction of the severity of caries in a rat model	

## 2 Polymers Attracting Calcium to Promote Remineralization

Ca and P are essential elements for the mineralization of both enamel and dentine. In natural oral environments, saliva is the primary source of calcium and phosphate ions. However, at carious sites with dental plaque, the excess phosphate in the plaque fluid results in the ratio of Ca and P falling below the optimal level required to promote remineralization, with a relative deficiency in calcium ions.^11,14^ Therefore, attracting sufficient calcium ions to demineralized enamel or dentine to promote remineralization represents a straightforward strategy, where polymers capable of binding calcium ions are utilized either as pretreatment agents on tooth surfaces or incorporated into other dental agents.

Dopamine, with catechol and amine groups, structurally mimics 3,4-dihydroxy-l-phenylalanine (DOPA) and lysine found in adhesive proteins of mussels. The self-polymerization of dopamine generates polydopamine (PDA), which could not only form adhesive coating on versatile substrates but also chelate cations due to abundant catecholamine moieties.^15,16^ Zhou et al. coated PDA on acid-etched enamel and dentine before immersion of the dental tissues in a supersaturated solution of calcium and phosphate.^17^ A marked promotion of dentine remineralization was observed with complete occlusion of dentinal tubules by densely arranged HA crystals. As for enamel, although polydopamine coating showed no statistically significant enhancement in enamel remineralization, the scanning electron microscopy (SEM) demonstrated a greater tendency of precipitate clusters to bundle in parallel and improved crystal packing density. In this study, PDA coating increased the quantities of calcium ions on the enamel and dentine surfaces due to the rich catecholamines of PDA and therefore promoted the formation of HA crystals. In addition to calcium binding, the mechanism of PDA in facilitating dentine remineralization was further investigated by Qu et al. from the perspective of interfacial control.^18^ PDA reduced the interfacial energy between collagen and amorphous calcium phosphate (ACP), which is the precursor to HA with liquid-like property, and therefore, the nanoprecursors acquired an easier access into the collagen fibrils. In the meantime, the decrease in the nucleation energy barrier expedited the phase transformation from an amorphous precursor to HA crystals.

It should be noted that treatment with PDA alone fails to induce remineralization of demineralized dental tissues, as it necessitates additional supersaturated calcium phosphate solutions to provide calcium ions.^17^ Saliva inherently serves as an abundant physiological reservoir of calcium and phosphate ions in the oral environment, and based on this, some polymers attracting intraoral calcium and binding demineralized tissues simultaneously are utilized to induce crystal nucleation and growth. For example, tannic acid (TA) was functionalized with a peptide sequence from statherin—one of the salivary-acquired pellicle (SAP) proteins.^19^ TA, though chemically classified as an oligomeric polyphenol rather than a true polymer, can undergo polymerization triggered by factors such as alkaline conditions, oxidation, and metal ions. It plays a dual role in the following system: (1) Its abundant pyrogallol groups enable efficient chelation of Ca^2+^ from saliva, facilitating HA crystal nucleation and growth. (2) TA coordinates with ferric ions (Fe^3+^) to form a stable, cross-linked coating (SAP-TA/Fe­(III)), which enhances its adhesion to acid-etched enamel surfaces. While TA alone lacks specificity for HA adsorption, conjugation with the peptide sequence—mimicking the enamel-binding domain of salivary statherin—enables targeted binding to demineralized enamel. Another example is P_11_-4, a self-assembled peptide which has already been commercialized.^20–22^ The solution of the peptide monomer permeates underneath the lesion through the pores in demineralized tissues, followed by the self-assembly and attraction of Ca^2+^ in saliva for nucleation. The results of clinical trials indicate the superior remineralization efficacy of P_11_-4 on buccal white spot lesions (WSLs) compared to applying fluoride varnish alone.^22^ However, the disadvantages of such methods are obvious, that the generated crystals around the peptide were arranged in a fan shape instead of in parallel, and the saliva-driven process was highly dependent on the amount and quality of saliva.

Chitosan, a biocompatible cationic polysaccharide, could also function as a pretreatment agent to enhance remineralization efficacy. Zhang et al. investigated chitosan’s role in a clinically relevant model incorporating a salivary pellicle. Their findings revealed that chitosan pretreatment, applied before a bioactive glass (BAG)-based remineralizing agent, significantly enhanced subsurface remineralization despite the presence of the pellicle barrier.^23^ Positive charges of chitosan facilitated strong adhesion to the negatively charged demineralized enamel surface, potentially enabling penetration into the lesion subsurface. Meanwhile, chitosan could coordinate with calcium ions from BAG due to its functional groups, forming complexes that promote apatite nucleation and growth within the lesion.^24,25^

Poly­(acrylic acid) (PAA) also acts as a qualified chelator for calcium ions due to its rich carboxyl groups and anionic nature at neutral pH. Milly et al. incorporated PAA into BAG air-abrasion powder and the PAA-BAG powder was subsequently used as a preconditioner for enamel WSLs before remineralization therapy.^26^ PAA-BAG removed a minimal enamel layer and increased surface roughness, thereby promoting deeper penetration of the remineralizing agents. The preconditioned lesions exhibited superior mineral deposition and microhardness, along with reduced subsurface porosity, indicating improved remineralization of WSLs. The role of PAA in apatite crystal formation was further elucidated in an evaporation-based strategy to synthesize centimeter-scale, enamel-like fluorapatite (FAP) sheets.^27^ The negatively charged low-molecular-weight PAA (LPAA) could adsorb onto FAP crystals and subsequently capture calcium ions from the solution to induce additional nucleation sites. Under LPAA-induced continuous nucleation, the resulting crystals were densely packed without intercrystalline gaps. Furthermore, the free carboxyl groups of LPAA reduced the interfacial energy between adjacent FAP layers, eliminating boundaries between them, which potentially contributed to superior mechanical properties. Notably, LPAA also demonstrated bactericidal efficacy against *Streptococcus mutans*, highlighting the significant potential of FAP-LPAA as a filling material for caries management.

## 3 Enamel Proteins and Mimics

### 3.1 Amelogenin and Amelogenin-Derived Peptide

The enamel matrix proteins crucially play regulatory roles throughout the mineralization process of enamel formation, with amelogenin standing as the most abundant type.^28^ Amelogenin mediates the nucleation of calcium phosphate, stabilization of mineral precursors, inhibition of premature crystal transformation, and oriented growth and morphologies of final crystals.^29–32^ Recombinant full-length amelogenins were utilized for early studies in mechanisms of amelogenin-involved mineralization and application for tooth remineralization, and for example, Fan et al. used one of the recombinant full-length porcine amelogenins, rP172, for biomimetic remineralization of acid-etched enamel, particularly in synergy with fluoride.^33^ Under physiological conditions, rP172 mediated the microstructure and organization of the newly formed fluoridated hydroxyapatite (FHA) mineral layer in a dose-dependent manner. The concentration of rP172 at 33 μg/mL initiated the formation of bundles of oriented FHA in a needle-like shape, and the higher concentration (i.e., 70 μg/mL) enhanced the crystal alignment and packing density.

In addition to full-length amelogenin, cleaved or spliced fragments of amelogenin may also retain essential capabilities comparable to the intact protein, considering that the functions of amelogenin predominantly originate from three amino acid segments: the hydrophilic C-terminus, the proline-rich central domain, and the hydrophobic tyrosine-rich N-terminus.^34,35^ Furthermore, the inherent complexity and high production costs of amelogenin synthesis have driven the adoption of amelogenin-derived peptides for biomimetic mineralization. Leucine-rich amelogenin peptide (LRAP), produced from alternative splicing during amelogenesis, comprises both N- and C-terminal domains and bears striking resemblances to amelogenin in the peptide assembly and regulation of mineralization in vitro.^36^ When applied to acid-etched enamel along with pyrophosphate-stabilized supersaturated calcium phosphate solution, LRAP selectively promoted crystal growth along the *c*-axis while modulating the crystal morphology which resembled the patterns of enamel rods.^34^ Furthermore, when utilized as a primer of surface treatment, LRAP presented remineralization capabilities for both superficial and deep enamel lesions.^38,39^ Another peptide, tyrosine-rich amelogenin peptide (TRAP), is the final proteolytic product of amelogenin obtained from the traces of a protein matrix after enamel maturation.^40^ In vitro studies on remineralization of enamel demonstrate that TRAP not only reduced the depth of lesions with gain of minerals but also regulated the morphology of HA crystals on the eroded surface of enamel.^41,42^ In addition to LRAP and TRAP, inspired by the conserved glutamine (Gln)-proline (Pro)-X repeat sequence of amelogenin, a 22-amino acid peptide, later named QP5, was designed combining five tandem repeats of Gln-Pro-X with the hydrophilic domains of the C-terminus.^43^ Beyond in vitro evidence of promoting enamel remineralization, the in vivo efficacy of this peptide has also been validated in a *S. mutans*-infected rat model, with enhanced mineral gain and reduced caries progression, performing comparably to sodium fluoride (NaF).^44^ Moreover, synergistic enhancement was observed when QP5 was combined with fluoride therapy, yielding a 38% increase in remineralized enamel microhardness.^42^ Recent research on its mechanism unveils that QP5 functions differently in solution or in the presence of enamel, as shown in Figure 1(A).^46^ In solution, spontaneous nucleation is inhibited because QP5, featuring its central (QPX)­5 domain and C-tail domain, adsorbs onto the mineral phase and stabilizes the ACP. However, on demineralized enamel surfaces, QP5 with its amino acids within C-tail negatively charged showed a preference for the HA (001) face via electrostatic interactions, followed by recruitment of calcium ions and consequently guiding the nucleation, growth, and orientation of HA crystals. This potential mechanism is not limited to QP5 but provides critical insights for other amelogenin-derived peptides such as QP5 in applications of enamel biomimetic mineralization.

**Figure 1:**
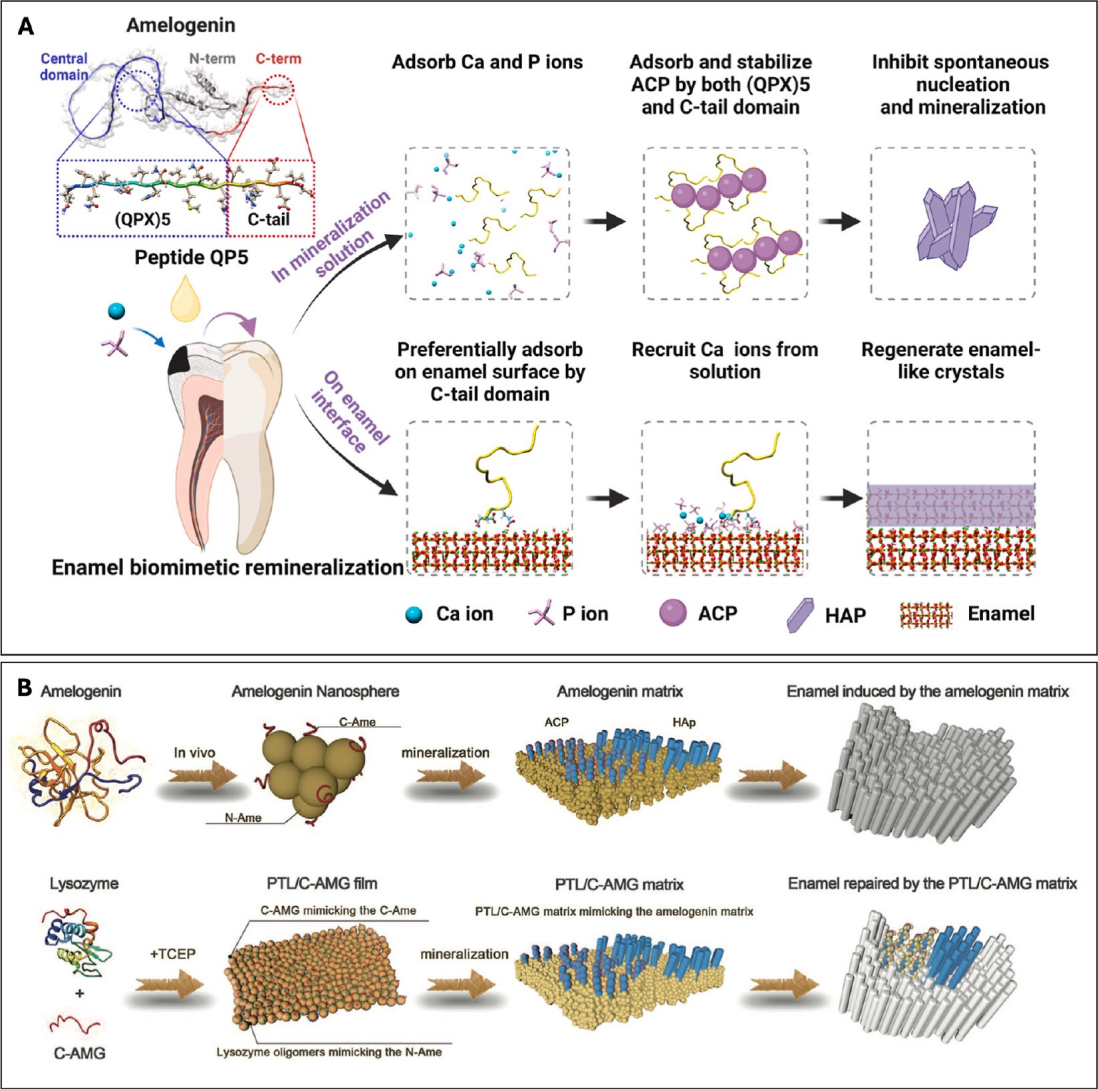
(A) Schematic demonstration of the two different mechanisms of QP5 in mineralization solution and on the enamel interface. Reprinted with permission from ref 46. Copyright ^©^ 2023 ELSEVIER. (B) Schematic demonstration of the amelogenin and PTL/C-AMG mediating the mineralization process. Reproduced with permission from ref 47. Copyright ^©^ 2020 WILEY.

While these peptides are derived from amelogenin, they cannot fully replicate the functions of amelogenin and thus have limited control over the formation of HA crystals, which still differ from native enamel. Therefore, scientists further seek to mimic the entire functions of amelogenin, especially including the highly conserved domains of the N- and C-termini, in a relatively low-cost and facile way without using the full-length amelogenin protein. As such, Wang et al. combined a phase-transited lysozyme (PTL) film and a synthetic peptide (C-AMG) to construct a biocompatible matrix (PTL/C-AMG) on enamel through a one-step coating strategy.^47^ It is schematically shown in Figure 1(B) how PTL/C-AMG mimics amelogenin behavior and the regulation of in situ remineralization. By reduction of the intramolecular disulfide bonds of lysozyme, it rapidly undergoes phase transition to form a film of oligomeric nanoparticles rich in β-sheets which emulates the amyloid-like aggregation characteristics of the N-terminal domain, providing templates for nucleation and strong adhesion to various substrates. The peptide C-AMG was synthesized according to the C-terminal domain and immobilized into the matrix for regulating the orientation and transformation of ACP. The nanofilm formed within 1 min and adhered robustly to the demineralized enamel surface, with the incorporated phosphate ions promoting the formation of ACP precursors. More importantly, the guiding effect of C-AMG resulted in epitaxial growth of HA along the *c*-axis and eventually highly ordered structures. Furthermore, the hardness of newly formed HA even exceeded that of native enamel, while the interfacial bonding strength between the mineralized layer and the substrate was superior to conventional fluoride treatment. Crucially, this strategy is available for nonmineralized substrates (e.g., silicon and copper) without the requirement for nucleation sites provided by residual crystal seeds and can be conducted through rapid coating (e.g., mouth rinsing), which suggests great applicability and potential for dental treatment.

### 3.2 Polymers to Stabilize Calcium Phosphate

According to the principles of nonclassical crystallization theory, the formation of natural enamel can be elucidated through a series of sequential steps. Initially, calcium and phosphorus ions coalesce to generate ACP. Subsequently, amelogenin functions to stabilize these ACP aggregates into clusters. Eventually, the ACP undergoes directional arrangement, culminating in the formation of HA bundles, which progressively evolve into enamel crystals and ultimately give rise to enamel prisms.^48^ To emulate the natural enamel crystallization process and facilitate the remineralization of demineralized enamel, it is essential to stabilize ACP such as amelogenin and promote directional and orderly crystallization of ACP into HA with enamel-like characteristics. Polymers such as casein phosphopeptides (CPPs), poly­(amidoamine) (PAMAM), polyelectrolytes, and chitosan derivatives can serve as stabilizers for ACP. An additional critical point is that in the nonclassical crystallization theory, it is prenucleation clusters (PNCs) that precede ACP formation. Like ACP, PNCs are also inherently unstable, and thus if utilized as the remineralization agent, they also require stabilizers to prevent rapid phase transformation.^49,50^

#### 3.2.1 Natural Polymers

CPPs, as phosphorylated peptides derived from casein hydrolysis, are enriched with acidic residues, particularly the –S _(p)_ S _(p)_ S _(p)_ EE-, which enables high-affinity interactions with calcium and phosphate ions under acidic conditions and formation of CPP-ACP or CPP-amorphous calcium fluoride phosphate (ACFP) complexes.^51^ CPP-ACP acts as a bioactive reservoir of mineralizing ions, and in the oral environment, the dissociation of calcium phosphate clusters could occur due to the strong HA-affinity of CPP and flow of saliva, followed by phase transformation to HA.^52^ CPP-ACP and CPP-ACFP have been commercialized as additives to dental products, such as Tooth Mousse and Tooth Mousse Plus. Substantial evidence-based research confirms that CPP-ACP serves as an effective calcium-phosphate-based agent capable of promoting enamel remineralization for caries management.^53^

Chitosan is remarkably biocompatible, biodegradable, nontoxic, and antimicrobial, but its practical application in multiple fields is constrained by limited water solubility caused by the inherent crystalline rigidity of the polymer structure. Carboxymethyl chitosan (CMC), synthesized through carboxymethylation of chitosan, stands as a water-soluble derivative exhibiting enhanced functional properties.^54^ Notably, compared to chitosan, the introduction of carboxyl groups strengthened the binding capacity of CMC for calcium ions.^55^ In the study of Xiao et al., CMC successfully stabilized ACP, forming CMC/ACP nanocomplexes.^56^ NaClO was then used to degrade the nanocomplex, breaking the combination of CMC and ACP to trigger the transformation from ACP to HA crystals. Finally, a chimeric peptide mimicking the functions of the C-terminal domain of amelogenin was applied to guide the crystals in an orderly manner. With these three steps, enamel-like crystals with great mechanical properties were generated, demonstrating the potential of this strategy for enamel remineralization. Similarly, Wang et al. selected CMC along with alendronate (ALN) as a stabilizer for ACP, and NaClO was used to eliminate the conjugation between CMC and ALN with generation of HA@ACP core–shell nanoparticles. After guidance with glycine, well-ordered crystals were formed on a demineralized enamel surface, realizing oriented biomimetic mineralization.^57^ Chen et al. used CMC to stabilize ACP as well as gold nanoparticles (AuNPs).^58^ The synthesized nanohybrids CMC/AuNP/ACP played a dual role: *S. mutans* was killed, attachment of biofilm was inhibited, and remineralization of demineralized enamel was promoted with superior performance to fluoride and CMC/ACP alone.

In addition to CMC, phosphorylated chitosan, as another derivative, also emerged as an analogue of natural phosphorylated proteins for biomimetic enamel remineralization. In the study of Zhang et al., phosphorylated chitosan (Pchi) acted as a stabilizer for ACP, forming nanoscale Pchi-ACP, and facilitated the subsequent remineralization of enamel lesions.^59^ In addition, the negatively charged Pchi enabled the adsorption of Pchi-ACP onto the positively charged sites of the demineralized enamel surfaces. Once adsorbed, Pchi-ACP released calcium and phosphate ions, promoting the repair of existing HA crystals and undergoing in situ transformation into new HA.

Starch is a type of soluble polysaccharide and has been used as a template for metal nanoparticles.^60,61^ In the study of Zhou et al., starch was used as a removable organic template to synthesize calcium phosphate prenucleation clusters (CaP–PNCs) for tooth repairing.^62^ On one hand, starch forms a gelatinized matrix that spatially confines and disperses calcium and phosphate ions, preventing aggregation and crystallization. On the other hand, the gelatinized starch, composed of amylose and amylopectin, provides hydroxyl groups that interact with calcium ions, enhancing the stability of the clusters while maintaining the amorphous phase. Notably, the CaP–PNC/starch complex exhibits long-term stability through freeze-drying, enabling storage at room temperature for up to five months without structural degradation. However, such a stable complex would fail to promote mineralization unless the organic template was removed, thus releasing CaP–PNCs for further transformation to ACP and crystals. Inspired by the natural process of mineralization in which protease is secreted to hydrolyze the proteins that inhibit CaP cluster nucleation, α-Amylase was applied to degrade the starch template, thereby eliminating the inhibitory effects on the CaP precursor to initiate on-site mineralization, as shown in Figure 2(A). This enzymatic removal of starch facilitates the transformation of CaP–PNCs into enamel-like hydroxyapatite structures, achieving efficient remineralization of both enamel and dentine. The starch template’s biocompatibility, cost-effectiveness, and precise enzymatic controllability underscore its superiority over conventional organic matrices, offering a clinically viable pathway for biomimetic material design and dental tissue repair.

**Figure 2:**
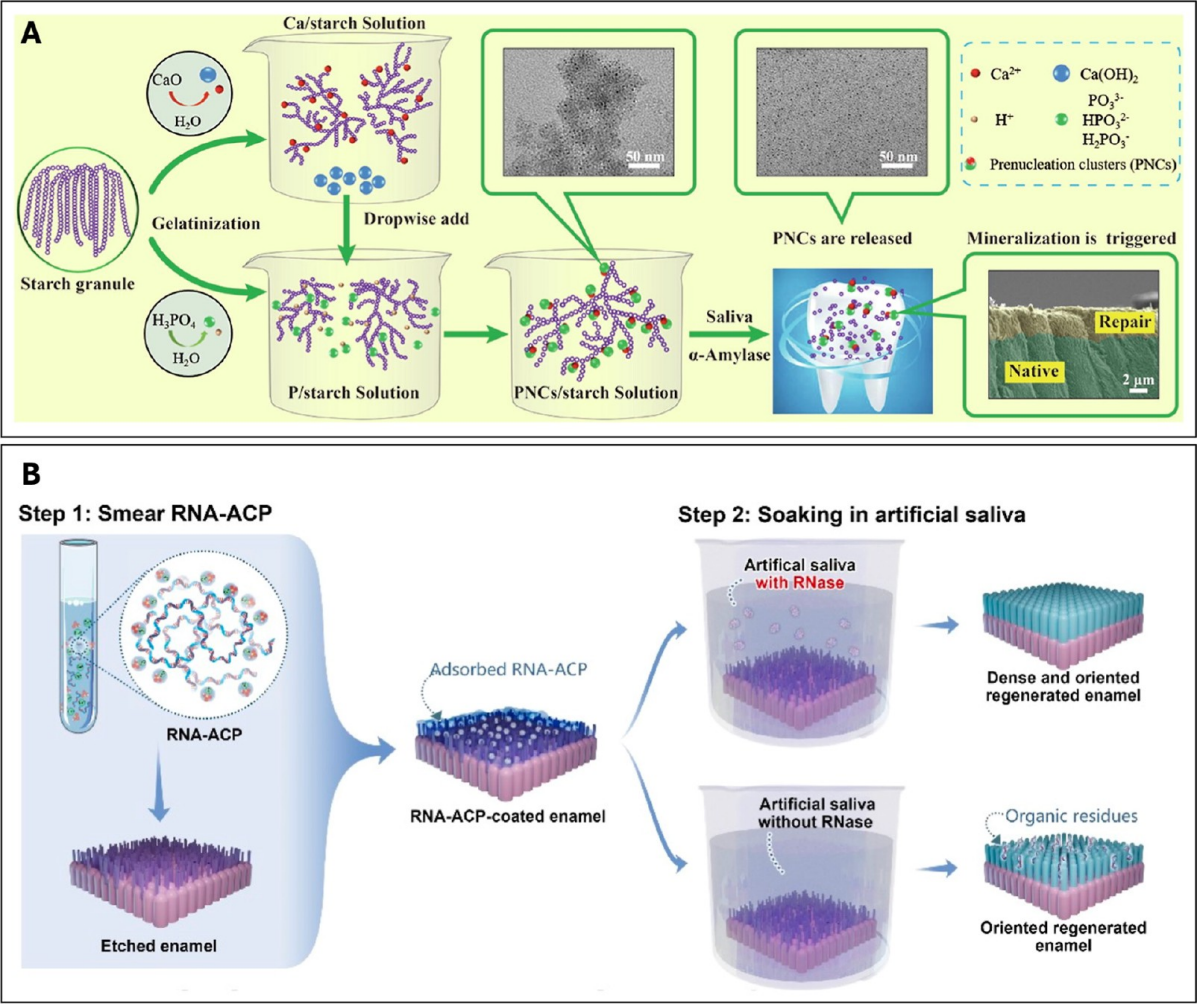
Two natural polymers to stabilize CaP, followed by degradation with specific enzymes. (A) Schematic illustration of starch stabilizing PNCs which were released in saliva containing α-Amylase and triggered mineralization. Reproduced with permission from ref 62. Copyright ^©^ 2021 ELSEVIER. (B) Schematic illustration of RNA-ACP applied to enamel and soaked in artificial saliva with or without RNase, exerting different effects on enamel mineralization. Reproduced with permission from ref 63. Copyright ^©^ 2024 WILEY.

As a natural polyanionic polymer with abundant phosphate groups, biocompatible RNA has also garnered increasing attention in biomimetic mineralization. Figure 2(B) schematically shows the study of Lei et al., which introduces a biomimetic strategy for enamel repair inspired by natural enamel maturation.^63^ The system combines RNA-stabilized ACP and RNase to achieve organic-free, dense enamel-like apatite regeneration. The negatively charged RNA interacted electrostatically with Ca^2+^ ions, enabling the formation of spherical RNA-ACP nanoparticles. The small size and the high affinity to enamel allowed adhesion and penetration into demineralized enamel pores, allowing epitaxial crystal growth with structural continuity between the repaired and natural enamel ensured. RNase, which naturally exists in saliva, triggered RNA degradation, initiating a process such as the maturation stage in amelogenesis. At this stage, organic templates will be degraded by relevant enzymes, and similarly, RNA degradation excludes organic residues from the mineral matrix, enabling lateral crystal growth and densification with enhanced mechanical and physicochemical properties of the repaired enamel.

#### 3.2.2 Synthetic Polymers

Synthetic polymers such as polyelectrolytes could also function as stabilizers for ACP, contributing to enamel remineralization. One of the commonly used polyelectrolyte stabilizers for ACP is PAA, which is rich in abundant carboxyl groups with great competence in binding calcium. In the study by Wang et al., 25 wt % PAA-ACP nanoparticles and 1 wt % sodium fluorescein were incorporated into a self-etch adhesive to develop a novel fluorescent mineralizing adhesive.^64^ ACP was maintained in a metastable state by PAA initially and transformed to nanocrystals, followed by assembly into HA under the regulation of PAA. Beyond promotion of mineralization, sodium fluorescein rendered the adhesive detectable without aesthetic compromise and cytotoxicity. The remineralization effect of PAA-stabilized ACP on WSLs was also investigated. Amine-functionalized mesoporous silica nanoparticles (aMSN) were selected as carriers for PAA-ACP, and the delivery system noted as PAA-ACP@aMSN.^65^ In vitro evaluation of artificial enamel WSLs revealed that this system achieved remineralization levels comparable to CPP-ACP.

First introduced by Tomalia et al. in 1985, PAMAM dendrimers feature a three-dimensional dendritic architecture with abundant amide groups and have been extensively applied in biomedical research due to diverse biological effects.^66^ PAMAM was imparted with the ability to facilitate mineralization after modification with carboxyl groups. PAMAM-COOH functions effectively as an organic template on the surface of demineralized enamel.^67,68^ In the presence of calcium ions, PAMAM-COOH underwent a stepwise self-assembly process closely resembling that of amelogenin, with the carboxyl groups acting as binding sites for calcium ions, and thereby stabilizing ACP.^69,70^ It facilitates the rapid formation of HA crystals that closely mimic the structural, orientational, and mineral phase characteristics of intact enamel.^67^ In addition to carboxyl-modified PAMAM, grafting PAMAM with other functional groups such as amines and hydroxyls could also bring improved binding with Ca^2+^ and adsorption to the enamel surface. Notably, PAMAM-NH_2_ and PAMAM-COOH effectively inhibited *S. mutans* adhesion through the formation of smooth, bacterium-resistant remineralized layers, which supports its application for carious tissues.^71^ In addition, the positively charged PAMAM-NH_2_ binds to the negatively charged bacterial cell membrane through the abundant amino groups on its surface, which may have a certain inhibitory effect on bacteria.^72^

Polyzwitterion has emerged as a novel and bifunctional agent with both remineralization and antibacterial efficacy in combating caries.^73^ As Figure 3 shows, poly­(carboxybetaine acrylamide) (PCBAA), with quaternary ammonium groups (positively charged) and carboxyl groups (negatively charged), provided binding sites for Ca^2+^ and PO_4_^3–^ simultaneously, strongly suppressing supersaturation of calcium phosphate and delaying ACP crystallization, which was evidenced by the maintained stability of the PCBAA/ACP nanocomposite for at least 3 days with no aggregation. It was demonstrated that PCBAA/ACP outperformed fluoride in promoting enamel remineralization both in vitro and in vivo. More than a stabilizer, PCBAA had potent antibiofilm functionality. Under physiological pH, its hydration and antifouling nature drastically reduced the initial adhesion of cariogenic bacteria like *S. mutans* to enamel. Under the acidic conditions caused by bacteria, the quaternary amino groups became cationic and endowed PCBAA with significant bactericidal activity, effectively killing adhered bacteria.

**Figure 3:**
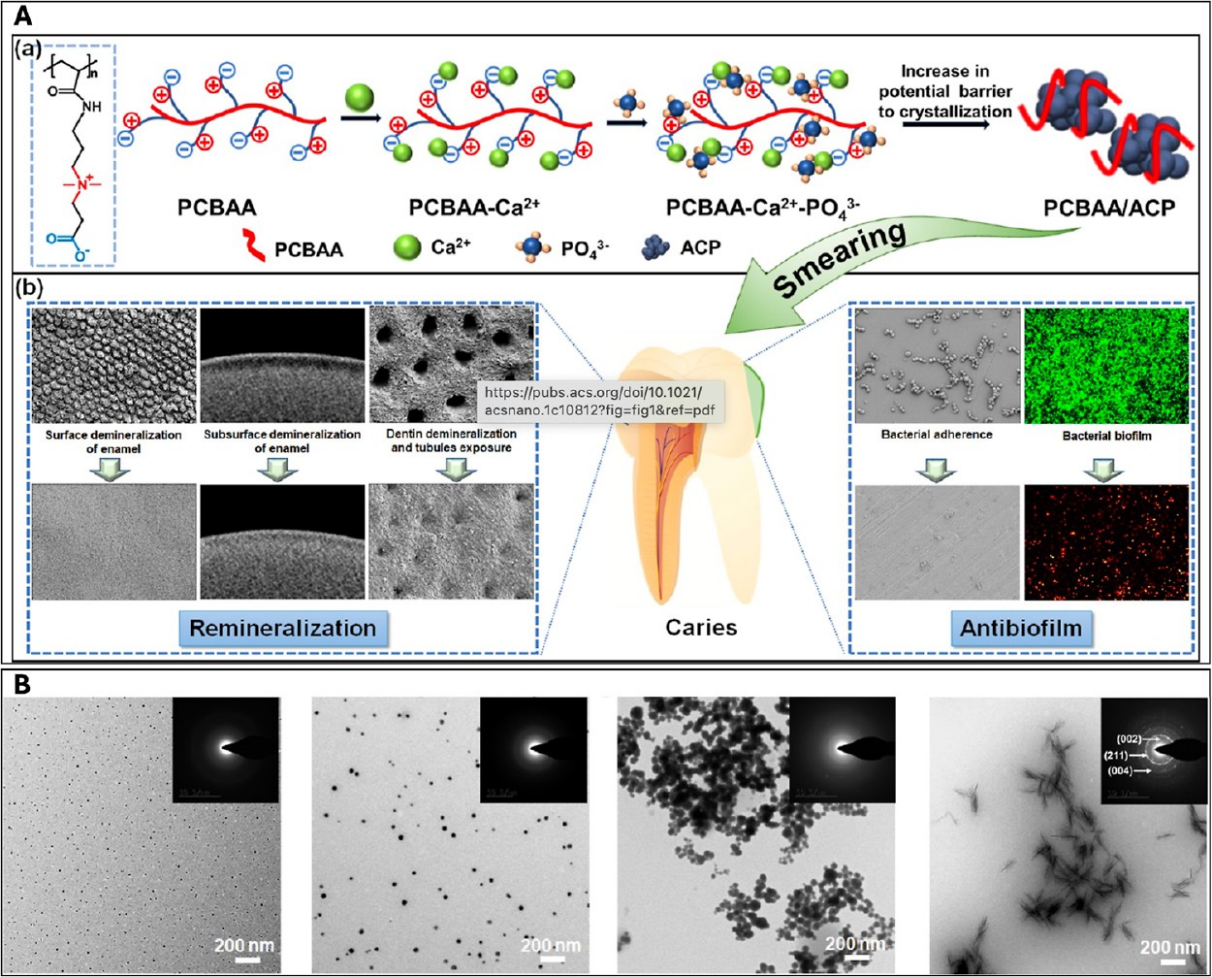
(A) Schematic diagram of the structure and dual functions of PCBAA/ACP. (B) TEM and SAED showing the transition of calcium phosphate. From left to right are the images of 1d, 3d, 5d, and 7d in sequence. On the 3rd day, no aggregation is observed, while on the 5th day, the amorphous state remained but with aggregation. Reproduced from ref 73. Copyright ^©^ 2022 American Chemical Society.

However, the aforementioned polyelectrolytes mimic certain functions of amelogenin primarily through their functional groups, while amelogenin proteins are internally disordered, which undergo intermolecular assembly when Ca^2+^ exists, forming functional β-sheet-rich fibrillar architectures.^74,75^ Hasan et al. employed elastin-like recombinamers (ELRs) to construct a supramolecular protein matrix that closely replicates the structural and functional characteristics of the native amelogenin matrix.^76^ Crucially, this synthetic matrix not only stabilizes ACP precursors but also directs the epitaxial growth of apatite nanocrystals preferentially along the *c*-axis. The ELR coating induced the formation of a mineralized layer with a controllable thickness (2–10 μm) on the demineralized enamel surface. More importantly, it was capable of recreating complex microstructures including prismatic and aprismatic enamel and also generated enamel prism-like structures on exposed dentine. The newly grown layer restored the mechanical properties of native enamel, including hardness, modulus, and wear resistance, and demonstrated resilience to clinically relevant challenges such as toothbrushing abrasion, acid attack, and chewing and grinding. This study highlights the potential of rationally designed polymers that emulate not only the chemical composition but also the hierarchical self-assembly and interfacial guidance functions of the native enamel matrix, marking a notable advance toward clinically translatable enamel regeneration.

## 4 Noncollagenous Proteins (NCPs) and Analogues

The main organic component of dentine is collagen, which acts as a scaffold for mineralization, while the remaining organics—NCPs—play a regulatory role in dentine mineralization. These proteins are negatively charged due to amino acid residues (e.g., aspartic acid and glutamic acid) and posttranslational phosphorylation of serine, threonine, tyrosine, etc.^32^ Such an attribute enables NCPs to interact with calcium ions and stabilize the calcium phosphate precursor.

### 4.1 NCP-Derived Peptides

Among all types of NCPs, dentine sialophosphoprotein (DSPP), dentine matrix protein 1 (DMP-1), bone sialoprotein (BSP), osteopontin, and matrix extracellular phosphoglycoprotein (MEPE), all are members of the Small Integrin-Binding Ligand N-linked Glycoproteins (SIBLINGs) family, playing critical roles in biomineralization of dentine.^5^ However, extraction, purification, or storage of those NPCs remains a challenging technique, and therefore, it is an alternative method to designing and synthesizing polypeptides with specific functional sequences of those NCPs.

Dentine phosphoprotein (DPP), derived from cleavage of DSPP, represents the most abundant NCP in dentine and regulates the formation of HA, which is primarily attributed to its repeating sequences of aspartate–serine–serine (DSS). DSS, with high affinity for HA and strong interaction with calcium phosphate, enables the 8DSS peptide to improve the nanomechanical behaviors of acid-etched enamel and promote remineralization of demineralized enamel in an artificial lesion model in vitro.^77,78^ Meanwhile, with great binding capacity for the dentine collagen matrix, 8DSS peptides induced mineral deposition on both demineralized dentine surface and dentinal tubules, resulting in improved mechanical properties and remarkable occlusion of dentinal tubules.^79,80^

DMP-1, with capacity for binding calcium and affinity for collagen, can regulate crystal nucleation, crystal growth, and mineral formation.^81,82^ Synthetic polypeptides derived from DMP-1 with both collagen-binding and HA-binding domains were demonstrated to stabilize calcium phosphate clusters with relative promotion of dentine remineralization, showing HA formation in the groups of collagenase-challenged and native dentine but not in the group of fully demineralized dentine.^83^ Notably, acidic clusters intermolecularly assembled into a β-sheet template are critical for DMP-1 inducing nucleation. Therefore, Li et al. designed and synthesized ID8, showing calcium-responsive self-assembly and containing nearly 100% β-sheet. This peptide could not only promote the intrafibrillar mineralization of dentine collagen but also the remineralization of artificial enamel lesions as well.^84,85^

### 4.2 Synthetic Analogues of NCPs

In comparison with peptides, using synthetic polymers is a more convenient way without the need for design and synthesis and consequently more commonly used as NCP analogues in research on biomimetic mineralization. Due to the association between highly acidic proteins and biomineralization, simple polyelectrolytes, poly­(aspartic acid) (pAsp) and poly­(glutamic acid), have been selected to modulate crystal growth.^86^ In a process named polymer-induced liquid precursor (PILP) proposed by Gower, pAsp could stabilize the amorphous precursor of calcium carbonate in a liquid-like state.^86,87^ The role of pAsp as an NCP analogue for intrafibrillar mineralization was first confirmed by Olszta et al.^88^ Based on the PILP process, pAsp induced the formation of fluid-like ACP, which infiltrates the nanogaps between collagen fibrils via capillary action. Subsequently, these hydrated precursors lose water and crystallize within the fibrils to form ordered nanostructured HA crystals, resulting in highly mineralized collagen that closely resembles that in natural bone tissues. After practice in bone formation, pAsp was also applied for dentine remineralization in the PILP process. In the study of Burwell et al., poly- *l* -aspartic acid was added to the remineralization solutions for dentine as a polymeric process-directing agent.^89^ The PILP system facilitated time-dependent functional remineralization of the partially demineralized dentine. Not only were mechanical properties restored but also intrafibrillar minerals increased with greater crystallinity and orientation like normal dentine. Furthermore, Bacinoa et al. investigated the feasibility of integrating the PILP process into restorative treatment of dentine lesions.^90^ Two strategies of applying pAsp were investigated: (1) incorporating pAsp into resin-modified glass ionomer (RMGI) cement and (2) rehydrating air-dried dentine lesions with a concentrated pAsp solution prior to RMGI restoration. All of the groups with pAsp showed significantly decreased shrinkage of the demineralized region, improved integrity of the cement–dentine interface, and restored modulus and hardness. The rehydration method achieved the highest mechanical recovery, with nanomechanical properties comparable to healthy dentine within 2 weeks. Despite some undesirable discoveries, such as cement porosity and interfacial reaction layers, pAsp still exhibited potential in minimally invasive dentistry by structurally and functionally repairing carious dentine, offering a promising pathway for clinical translation of PILP-based therapies. In addition to remineralization of surface defects of dentine, the PILP strategy with pAsp was also feasible for occlusion of dentinal tubes.^91^ The pAsp-stabilized ACP system facilitated intrafibrillar mineralization of collagen, followed by epitaxial HA crystal overgrowth, resulting in a dense, compact mineral layer deeply occluding dentinal tubes and ensuring robust mechanical bonding between the mineralized layer and the organic matrix. This demonstrated that the potential clinical use of the PILP system was not limited to caries but dentine hypersensitivity as well.

PAA, negatively charged with abundant carboxyl groups, is also widely used as a synthetic analogue for the regulation of mineralization. Olszta first realized the intrafibrillar mineralization of type I collagen through the PILP process, but on calcium carbonate.^92^ However, as for the calcium phosphate system, PAA alone was considered unable to induce mineralization of collagen in the early years.^93^ Combination of PAA and polyvinylphosphonic acid (PVPA) in Portland cement/phosphate-containing fluid (PCF) was demonstrated to guide both interfibrillar and intrafibrillar remineralization of demineralized dentine.^94^ PAA functioned as a calcium-phosphate-binding analogue, stabilizing ACP and enabling their penetration into the collagen matrix. PVPA, mimicking phosphoproteins, anchored these stabilized precursors to the collagen scaffold, directing intrafibrillar and interfibrillar apatite nanocrystal deposition. The synergistic effect of PAA and PVA was critical in this system, as PAA alone produced ACP nanospheres with limited collagen infiltration, while PVPA alone lacked nucleation efficacy. Following research has discovered that it is some key parameters (e.g., concentrations and molecular weight) that have a great influence on the performance of PAA in promoting intrafibrillar mineralization. The study of Wang et al. demonstrated the dose-dependent relationship between PAA and ACP.^95^ Relatively low concentration (100 μg mL^–1^) allowed rapid ACP aggregation, resulting in superficial, disordered mineralization, while high concentration (1000 μg mL^–1^) impeded the phase transformation of ACP and subsequently dentine remineralization. At a moderate concentration of 500 μg mL^–1^, PAA optimally stabilized ACP in a liquid-like state, enabling its infiltration into collagen fibrils and transformation into hierarchical HA resembling natural dentine. The study of Qi et al. highlighted the dual role of PAA molecular weight (MW) and concentration in regulating collagen mineralization.^96^ Low-MW PAA (2 kDa) at high concentrations (50 mg/L) resulted in overly stabilized ACP precursors, halting mineralization entirely; while high-MW PAA (450 kDa) at low concentrations (10 mg/L) induced rapid crystallization, resulting in disordered surface mineralization. Intermediate combinations (50 kDa PAA at 25–50 mg/L) achieved hierarchical intrafibrillar mineralization, producing collagen–HA composites. These studies revealed that lower MW and higher PAA concentrations increased ACP stability, thus delaying aggregation and phase transformation, which, in turn, would be accelerated in the case of higher MW and lower concentrations. This not only provides guidance for PAA but also for other polymeric NCP analogues, that the MW and concentration should be simultaneously optimized to balance precursor stabilization and mineralization kinetics.

CMC with abundant carboxyl groups also mimics the function of DMP-1 like pAsp and PAA and acts as an ACP stabilizer. In the study of Chen et al.,^97^ CMC and the stabilized ACP formed CMC/ACP gel which was then processed into CMC/ACP scaffolds via lyophilization. The scaffold released ACP nanoparticles upon dissolution, facilitating intrafibrillar mineralization. CMC/ACP nanocomplexes penetrated the gap zones of collagen and enabled the deposition of hydroxyapatite within fibrils. Additionally, CMC’s biocompatibility, biodegradability, and antibacterial properties make it potentially suitable for further application in caries management.

In addition to anionic polyelectrolytes, cationic polyelectrolytes could also serve as potential analogues. Cantaert et al. first demonstrated that in the presence of a positively charged additive, i.e., poly­(allylamine hydrochloride) (PAH), the precipitation of calcium carbonate was strongly influenced with generation of calcite in similar morphologies as those produced with pAsp via PILP.^98^ Niu et al. demonstrated that PAH stabilized ACP and facilitated ACP infiltration into collagen fibrils, despite potential electrostatic repulsion in cationic collagen systems.^99^ Based on this, systems containing PAH-stabilized ACP were developed for mineralization of dentine. In the study of Yang et al., silica/mesoporous titanium–zirconium (STZ) was utilized to carry PAH-ACP, producing a yolk–shell nanocomposite named PSTZ.^100^ PAH, on one hand, maintained ACP in its amorphous phase and prevented early crystallization; on the other hand, it enhanced the efficiency of loading ACP due to electrostatic interactions with negatively charged STZ. The nanocomposite exhibited multiple functions including immediate occlusion of dentinal tubes, induction of intrafibrillar collagen mineralization, and stimulation of odontogenic differentiation of dental pulp stem cells. Yu et al. constructed a multifunctional nanosystem of hollow mesoporous silica loaded with epigallocatechin gallate (EGCG) and PAH-ACP.^101^ In this system, PAH-ACP preserved its capacity to induce collagen mineralization and also synergized with antimicrobial EGCG to achieve dual therapeutic effects (occluding dentinal tubules and suppressing cariogenic biofilms), thus offering a promising approach for managing dentine hypersensitivity and caries through control of both remineralization and biofilm.

As introduced before, modified PAMAM (e.g., PAMAM-COOH, PAMAM-OH, and PAMAM-NH_2_) could stabilize calcium phosphate and induce enamel remineralization. On top of that, these PAMAM dendrimers have also been explored as synthetic analogues of NCPs for dentine remineralization. According to Li et al., PAMAM-COOH, particularly the fourth generation (G4-COOH, ∼10 kDa), mimicked the dual functions of NCPs.^102^ Its carboxyl groups enabled the capture and stabilization of ACP, while its molecular size allowed for retention within the collagen fibril matrix. Consequently, immobilized G4-COOH acted as a template, directing the ordered assembly and crystallization of ACP precursors within the collagen fibrils and achieving intrafibrillar mineralization of dentine. Similarly, the third-generation PAMAM-NH_2_ (G3-PAMAM-NH_2_) with its MW around 6909 Da could penetrate intrafibrillar spaces and bind to the fibrils, acting as templates for ACP stabilization and transformation and inducing mineralization within type-I collagen.^103^ Furthermore, G3-PAMAM-NH_2_ was also demonstrated to effectively bind to demineralized dentine and induce formation of needle-like crystals which densely covered the intertubular dentine surface and deeply occluded dentinal tubules.^104^ Gao et al. compared the fourth-generation PAMAM-NH_2_ with NaF, a conventional desensitizing agent for dentine hypersensitivity.^105^ While NaF initially induced mineralization more rapidly, demineralized dentine treated with PAMAM and soaked in artificial saliva after 28 days achieved comparable reductions in dentine permeability. PAMAM-NH_2_ induced deeper intratubular mineralization, and therefore, the dentinal tubule occlusion demonstrated significantly superior acid resistance, suggesting higher stability.^104^ The fourth-generation PAMAM-OH also exhibited similar capacity for binding demineralized dentine and inducing occlusion of dentinal tubules; however, its remineralization effectiveness was inferior to that of PAMAM-NH_2_ and PAMAM-COOH which had stronger electrostatic interactions with collagen and potentially higher binding affinity for calcium ions.^106,107^ Notably, the unique structure of PAMAM allows further functionalization via external and internal modifications. As shown in Figure 4, PAMAM-NGV@galardin (PNG) was synthesized by externally grafting an NCP-derived peptide (i.e., NGV) on the carboxyl terminals of the fifth generation of PAMAM-COOH and internally loading a small molecular insoluble drug (i.e., galardin) in the hydrophobic cavity of PAMAM.^108^ In addition to the retained ability of PAMAM to induce dentine remineralization, NGV and galardin played a synergistic role in protecting collagen. NGV was responsible for restricting the movement of collagen and promoting collagen cross-linking, while galardin released from PAMAM for inhibiting matrix metalloproteinases (MMPs) which cause collagen degradation. PNG with combined remineralization and collagen-stabilization competence restored dentine structure and mechanical properties even under collagenase-rich conditions and showed anticaries effects in vivo.

**Figure 4:**
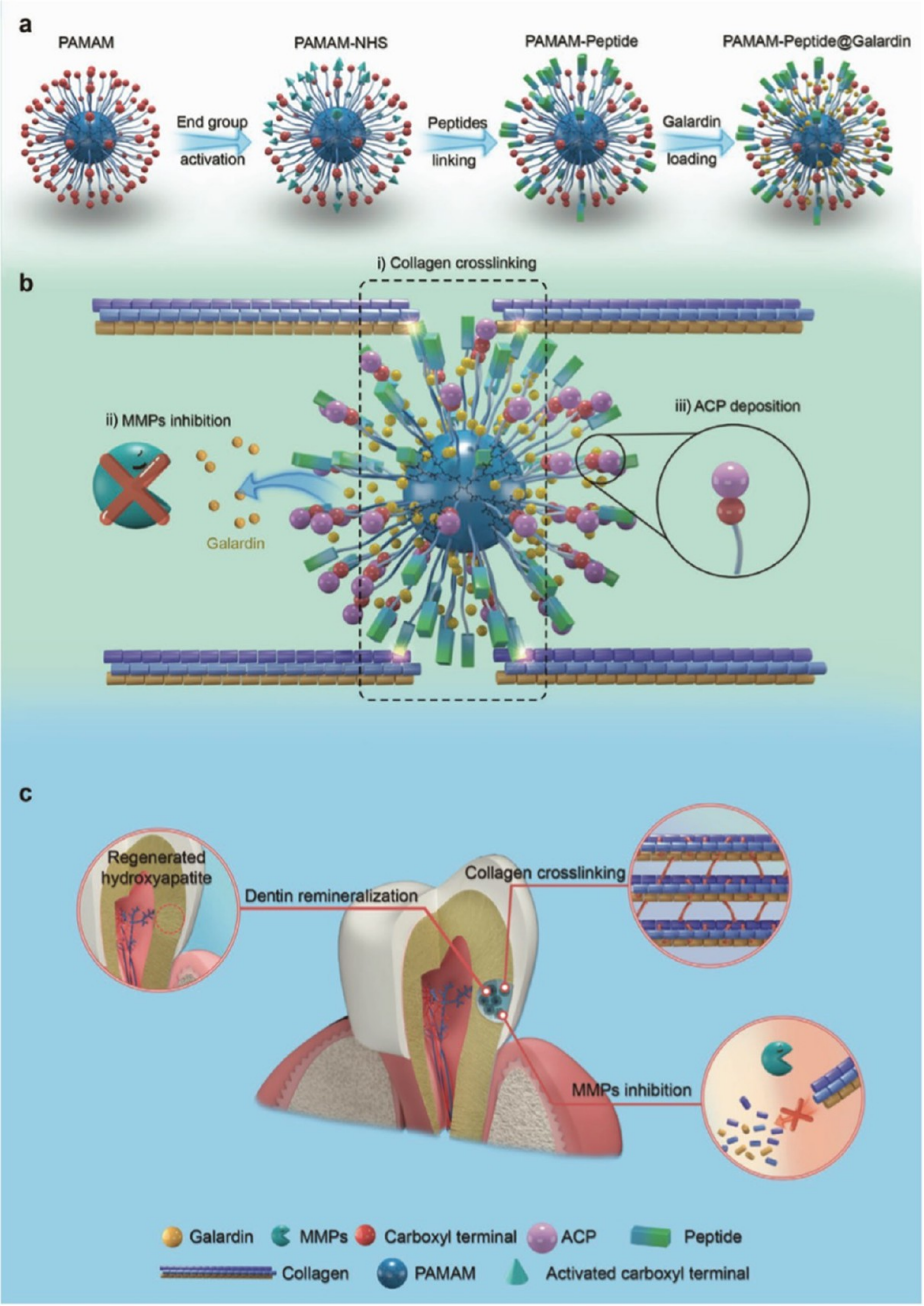
Schematic illustration of (a) PNG preparation; (b) mechanisms of multifunctional PNG for collagen cross-linking, ACP deposition, and MMP inhibition; (c) dentine repair via collagen stabilization and dentine remineralization. Reproduced with permission from ref 108. Copyright ^©^ 2022 WILEY.

Peptoids, a class of biomimetic N-substituted glycine polymers with sequence-specific side chains, possess great biological activities and functionalities.^109,110^ Although peptoids share structural similarities with peptides, they are not genuine peptides. Amphiphilic peptoids with alternating hydrophobic and hydrophilic residues could act as synthetic surrogates for NCPs.^111^ Their aromatic groups could bind collagen via CH–π interactions, which improved the assembly of the collagen matrix, while anionic carboxylate side chains regulated the calcium phosphate nucleation and growth. When pretreatment with peptoids and mineralization mediated by PILP were combined, the ultrastructure of peritubular dentine was notably restored. This was achieved by directing the alignment of HA nanocrystals along the peripheries of the tubules—an outcome that the PILP process alone or the addition of peptoids to PILP with pAsp failed to accomplish. Correspondingly, the mechanical properties were enhanced. The sequence-dependent efficacy of peptoids highlights their potential as customizable NCP mimics for functional tissue remineralization.

### 4.3 Natural Analogues of NCPs

Compared with the diversity of synthetic polymers as NCP analogues, research on natural polymers as NCP analogues remains scarce. Proanthocyanidin (PA), the polymer of flavan-3-ols originated from some natural plants, possesses great biological and biomedical potency and has been recently used to modify dentine due to its ability to inhibit MMPs and cross-link collagen.^112,113^ Recently, Chen et al. integrated PA into the PILP process.^114^ By forming hydrogen and hydrophobic bonds with collagen, PA cross-linked and stabilized the demineralized collagen matrix, preventing enzymatic degradation and maintaining its structural integrity. Simultaneously, PA facilitated the infiltration of ACP nanoparticles into collagen fibrils due to the fluidity of PA–ACP nanocomplexes, thus accelerating intrafibrillar mineralization. PA significantly reduced the remineralization time while achieving hierarchical mineral organization and mechanical properties comparable to natural dentine.

## 5 Polymers Providing Mineralization-Favorable Environment (in the Form of Hydrogel)

In the previous sections, various polymers have been discussed for their roles in inducing or promoting mineralization on enamel and dentin—either by attracting calcium ions or imitating the functions of amelogenin and NCPs. Beyond these polymers, hydrogels figure prominently in the biomimetic mineralization of enamel and dentine. The polymers involved in the hydrogel system facilitate mineralization mainly in two modes: First, as hydrogels are a class of three-dimensional polymeric materials composed of highly cross-linked, hydrophilic polymer chains,^115^ the construction of any hydrogel-based mineralization system intrinsically requires polymers as its structural foundation. Once hydrogels formed, they can act as versatile carriers that accommodate a wide range of cargos,^116^ including biomacromolecules (e.g., amelogenin and NCPs) and mineral ions for promoting mineralization ions. Second, some polymers form the gel network while also directly participating in the mineralization process through functional groups such as hydroxyl or carboxyl, attracting calcium and guiding the nucleation and growth of HA. Here, hydrogels are not merely perceived as a form of polymeric material but as an integrated system that transcends the function of individual polymers in enamel and dentine mineralization, providing multifaceted advantages for creating a mineralization-favorable environment at both physical and practical levels. Physically, the hydrogel mimics the gel-like environment during natural enamel and dentine development,^117,118^ traps ions and molecules to localize mineral sources and templates, and confines reactions to the hydrogel–tissue interface. Practically, the hydrogel possesses the following capabilities: presenting swellability, biocompatibility, biodegradability, mucoadhesiveness, injectability, and in some cases, in situ gelling; isolating interfering factors like saliva and food debris if applied in real oral cavity;^119^ and the flexibility to incorporate any additional functional substances to endows the system with anticaries properties and so on. Collectively, these features grant hydrogel-based mineralization systems clinical operability and translational potential, distinguishing them from other polymeric strategies.

In this section, hydrogel-based mineralization strategies will be categorized based on the functional modes in which polymers facilitate mineralization. Polymers as the raw materials for hydrogels carrying the mineralization-promoting agent will be first introduced. Subsequently, dual-functional polymers, serving simultaneously as both network formers and mineralization templates, will be focused.

### 5.1 Polymers Forming Hydrogel as a Carrier

Collagen is a natural component of the extracellular matrix and the most abundant protein in dentine. Collagen hydrogels could serve as a natural scaffold or matrix model for research on collagen-based mineralization. For example, odontogenic ameloblast-associated protein (ODAM) and amelotin (AMTN) are two essential proteins secreted by ameloblasts at the maturation stage of amelogenesis.^120^ Ikeda et al. investigated the capacity of ODAM and AMTN for mineralization promotion in collagen hydrogel systems.^121,122^ ODAM promoted HA nucleation in a dose-dependent manner in simulated body fluid (SBF) and crucially induced mineralization within a collagen hydrogel matrix, forming surface HA deposits after 24 h; while AMTN embedded in collagen hydrogel induced rapid HA deposition both superficially and internally within just 5 h. The collagen served not only as a structural scaffold but also as a stable carrier, where AMTN incorporated into collagen gels showed minimal release (∼1%), attributed to potential cross-linking via agents like genipin.

Chitosan and its derivatives are described in the previous sections with functions as the pretreatment agent, amelogenin mimic, and NCP analogue. Since chitosan can be easily processed into hydrogels and chitosan-based hydrogels are widely used across various fields due to their biocompatibility and functionality, research on these hydrogels has also been extended to the remineralization of enamel and dentine.^123,124^ However, the chitosan hydrogel alone is unable to promote remineralization, so peptides or calcium phosphate agents are also usually incorporated into the hydrogel to form a mineralization system. Amelogenin-containing chitosan (CS-AMEL) was developed by Ruan et al., combining the advantages of chitosan and amelogenin.^125,126^ Primarily serving as a biocompatible, biodegradable, and clinically manageable hydrogel matrix, chitosan effectively carried and delivered amelogenin, which could stabilize CaP clusters and induce organized crystallization. Beyond this basic function, chitosan exhibited three other properties: (1) Antimicrobial property: Chitosan possesses an antibacterial ability against cariogenic bacteria. (2) pH-responsiveness: When the pH ranged between 5.0 and 5.5, chitosan’s amino groups attracted H^+^ ions, forming a barrier that could inhibit acid penetration and subsequent demineralization. Simultaneously, protonated amino groups electrostatically interacted with amelogenin, potentially protecting it. This interaction weakened, and amelogenin was released to perform its regulatory role when the pH returned to 6.3 and 7.0 due to saliva. (3) Adhesiveness: The mucoadhesive nature of chitosan would promote retention on the tooth surface. In another relevant study where CS-AMEL was evaluated in a pH-cycling model in vitro, the efficacy of the system was confirmed through an organized enamel-like layer formed on erosive lesions and regrowth of oriented crystals on artificial early caries with reduced lesion depth.^127^ Taken together, CS-AMEL stands as a promising hydrogel system for enamel repair and caries management. Similarly, QP5, an amelogenin-derived peptide, was also combined with chitosan hydrogel, with QP5 acting as a regulator for enamel remineralization and chitosan as an antibacterial, pH-responsive, adhesive carrier.^128^ The hydrogel system demonstrated significant effects on remineralization of enamel lesions after being challenged by pH cycles and also demonstrated antibacterial effect on *S. mutans* with adhesion and biofilm formation reduced over 95% within 24 h, and biofilm growth, lactic acid production, and metabolic activity inhibited within 7 days.^129^ Researchers have also incorporated calcium phosphate into the chitosan hydrogel to develop self-sufficient remineralizing systems. For example, nanohydroxyapatite (nHA) could be embedded within the chitosan hydrogel, and Fathy et al. found that the formulation with a higher chitosan content (70/30 HAp-CS) significantly improves cross-sectional microhardness and surface morphology in mildly demineralized lesions.^130^ In the study of Rafiee et al., the gel containing chitosan and nHA outperformed chitosan plus NaF and NaF varnish, while the combination of chitosan, nHA, and NaF showed the best remineralization effects.^131^

Notably, certain hydrogel systems are designed to form in situ in response to certain stimuli. Such in situ forming hydrogels are easier to handle and can better adapt to uneven tissue surfaces like demineralized enamel or penetrate into porous tissue like exposed dentinal tubules. Fletcher et al. incorporated ACP into poly­(vinylpyrrolidone) (PVP) nanofibers to build ACP/PVP electrospun mats which would turn into hydrogels on enamel upon hydration by fluoride-containing artificial saliva.^132^ Unlike the polyelectrolytes described in the previous sections, PVP did not stabilize ACP through specific functional group interactions. Instead, ACP nanoparticles were physically entrapped within the PVP matrix during electrospinning. Following hydration, the electrospun mats swelled to form a hydrogel, within which the ACP was retained as a localized reservoir of calcium and phosphate ions. When applied on acid-etched enamel, in situ transformation of ACP occurred and a continuous layer of fluoridated HA was generated. Furthermore, ACP/PVP also worked for dentine remineralization, as its hydrogel state allowed infiltration into exposed dentinal tubules, causing occlusion. In the study by Rafiee et al. mentioned in the previous paragraph, the chitosan hydrogel was also fabricated as an in situ forming system and was thermoresponsive. At room temperature, it existed as a low-viscosity solution, which facilitated storage and local administration. It transformed into a gel upon exposure to an artificial saliva environment at 37 °C.^131,133^

### 5.2 Polymers Forming Hydrogel as Both Mineralization Template and Carrier

In many recent studies on enamel and dentin mineralization, beyond simply serving as a raw material for hydrogels and playing a basic carrier function, many polymers themselves can also act as templates for mineralization. Gelatin, a type of denatured protein produced by collagen hydrolysis, has been widely exploited in the food industry and pharmaceutical industry.^134^ Busch developed a gelatin hydrogel-based mineralization system to generate enamel-like fluorapatite layers on human teeth.^135^ The system comprised four layers, i.e., etched enamel, phosphate/fluoride-enriched gelatin gel, a phosphate-free gelatin as a shielding layer to prevent calcium from precipitating with phosphate, and calcium solution. In addition to acting as a reservoir of mineral ions, gelatin with positively charged N-terminal amino groups also acted as an organic matrix to template the nucleation by binding to phosphate groups on the etched enamel surface. This interaction aligned gelatin polypeptides perpendicularly to the substrate, which in turn directed the epitaxial growth of FAP crystals parallel to the organic matrix, achieving a perpendicular orientation like natural enamel prisms. However, in this study, glycerine was used to modify the gelatin to increase its melting point to approximately 40 °C; otherwise, the gel would melt at physiological temperature in the oral cavity.

Agarose, with a higher sol–gel transition temperature (∼60 °C), overcame the limitation of gelatin, demonstrating great potential in enamel and dentine mineralization. Cao et al. constructed a similar four-layer system with etched enamel, a CaCl_2_-loaded agarose hydrogel, an ion-free agarose hydrogel, and a phosphate solution. The agarose controlled unidirectional diffusion of calcium and phosphate ions toward the etched enamel substrate, stabilized amorphous mineral precursors, and facilitated crystal growth along the *c*-axis perpendicular to the enamel surface and their subsequent parallel alignment into prism-like bundles.^136^ The application of agarose hydrogel in dentine mineralization was also demonstrated, resulting not only in the formation of enamel prism-like tissue on acid-etched dentine surfaces but also in the deep occlusion of dentinal tubules.^137,138^ Furthermore, an agarose-based hydrogel system was successfully employed in vivo in a rabbit model. Notably, the agarose gel accommodated itself to a custom tray for overnight application and exhibited stability in a complex oral environment, indicating potential in clinical repair of dentine lesions.^119^

In addition to calcium ions loaded by gelatin and agarose hydrogel, polymer-stabilized ACP could also be delivered by the hydrogel system. Hydroxypropylmethylcellulose (HPMC) is a cellulose derivative and semisynthetic polymer rich in hydroxyl, methyl, and methoxy groups.^139^ In the study of Wang et al., HPMC was utilized as the matrix for a mineralizing film or gel to deliver pAsp-stabilized ACP nanoparticles.^140^ More than a basic carrier, it served as a responsive platform and ACP stabilizer, enabling effective precursor delivery and controlled mineralization kinetics for dental hard tissue repair. This is attributed to its unique physicochemical properties: it formed stable dry films, making them convenient for storage and application while transforming into a hydrogel in the moist oral environment. This phase change contributed to the controlled release kinetics of the encapsulated pAsp-ACP nanoparticles. Furthermore, the hydroxyl and methoxyl groups of HPMC synergized with pAsp to stabilize ACP, significantly prolonging their bioactive state and preventing early crystallization into HA. The gel HPMC also provided a microenvironment beneficial for ion diffusion and electrostatic interactions between Ca^2+^ and polyhydroxyl of HPMC, contributing to sustained release of Ca/P and consequently the efficient mineralization results observed—repair demineralized dentine within 96 h.

Hydrogels as mineralization templates can also be involved in the PILP process. Elastin-like recombinamers (ELRs) are synthetic biopolymers designed and produced by using recombinant DNA technology, mimicking the structure of elastin. An essential feature of ELRs is their thermoresponsiveness, allowing a reversible phase transition that facilitates hydrogel formation above a specific transition temperature (Tt).^141^ In the study by Li et al., cross-linked ELR hydrogels served as synthetic organic templates for biomimetic mineralization via the PILP process.^142^ The microporous structure self-assembled by the ELRs provided a confined framework for the selective deposition of minerals. The PILP process was induced by pAsp, and fluidic ACP infiltrated deeply into the ELR matrix. This resulted in regulated mineral deposition, preserving the original microporous structure and creating a highly integrated organic–inorganic nanocomposite. A mineral density comparable to that of natural bone and dentine was achieved with superior mechanical properties. Therefore, ELRs offer a promising synthetic alternative to natural matrices like collagen for hard tissue repair.

The hydrogels mentioned above are made from a single polymer that serves both as the raw material and as the mineralization template. However, since hydrogels can be formed by the cross-linking of more than one polymer, this allows a single hydrogel system to incorporate multiple mineralization templates during fabrication. For example, Wen et al. fabricated an injectable mineralized hydrogel comprising three polymers: (1) PAA, as a mimic of NCPs, established a dynamic Ca^2+^·COO^–^ coordination network, endowing the hydrogel with self-healing and shear-thinning properties critical for clinical delivery; (2) CMC reinforced mechanical stability while modulating degradation kinetics and swelling behavior; and (3) treated dentine matrix (TDM) served as a bioactive mineralization template, directly stimulating odontogenic or osteogenic differentiation in dental pulp stem cells.^143^ In vivo, PAA-CMC-TDM promoted functional regeneration in complex bone defects and dentine–pulp interfaces. Although, strictly speaking, this study employs a cell-involved approach for dentine regeneration—distinct from the other cell-free remineralization strategies discussed in this review—the methodology used to construct mineralized hydrogels remains valuable for reference.

One of the greatest advantages of hydrogel systems lies in their excellent customizability, allowing the incorporation of various components on demand to achieve multifunctionality. Recent innovative hydrogel systems are no longer limited to promoting enamel and dentin mineralization. They also integrate antimicrobial and antibiofilm functions, thereby enhancing their potential for practical application in caries treatment. Ran et al. developed a multifunctional hydrogel named BP@CP5 consisting of catechol-modified chitosan (CHI-CS), PLGA-PEG-PLGA (PPP), and black phosphorus nanosheets (BPNs), as shown in Figure 5(A).^144^ CHI-CS enhanced the wet adhesion properties, due to its catechol groups facilitating binding with the tooth surface, which ensured that the hydrogel could remain in place even in the dynamic and moist environment of the oral cavity; while PLGA-PEG-PLGA imparted injectability and temperature-sensitivity to the hydrogel. This polymer would self-assemble into core–shell micelles in aqueous solutions and form a gel at body temperature. As such, this temperature-sensitive behavior made the hydrogel easily applied in a liquid form and then solidified at around 37 °C, providing a stable and long-lasting barrier on the tooth surface. After irradiation, attributed to the photothermal activity of BPNs, the system exerted bactericidal effects on *S. mutans* and *Streptococcus sanguinis* and generated phosphate ions expediting enamel remineralization. Furthermore, this hydrogel system demonstrated excellent performance on caries prevention in a rat model, suggesting the potential of BP@CP5 for caries management.

**Figure 5:**
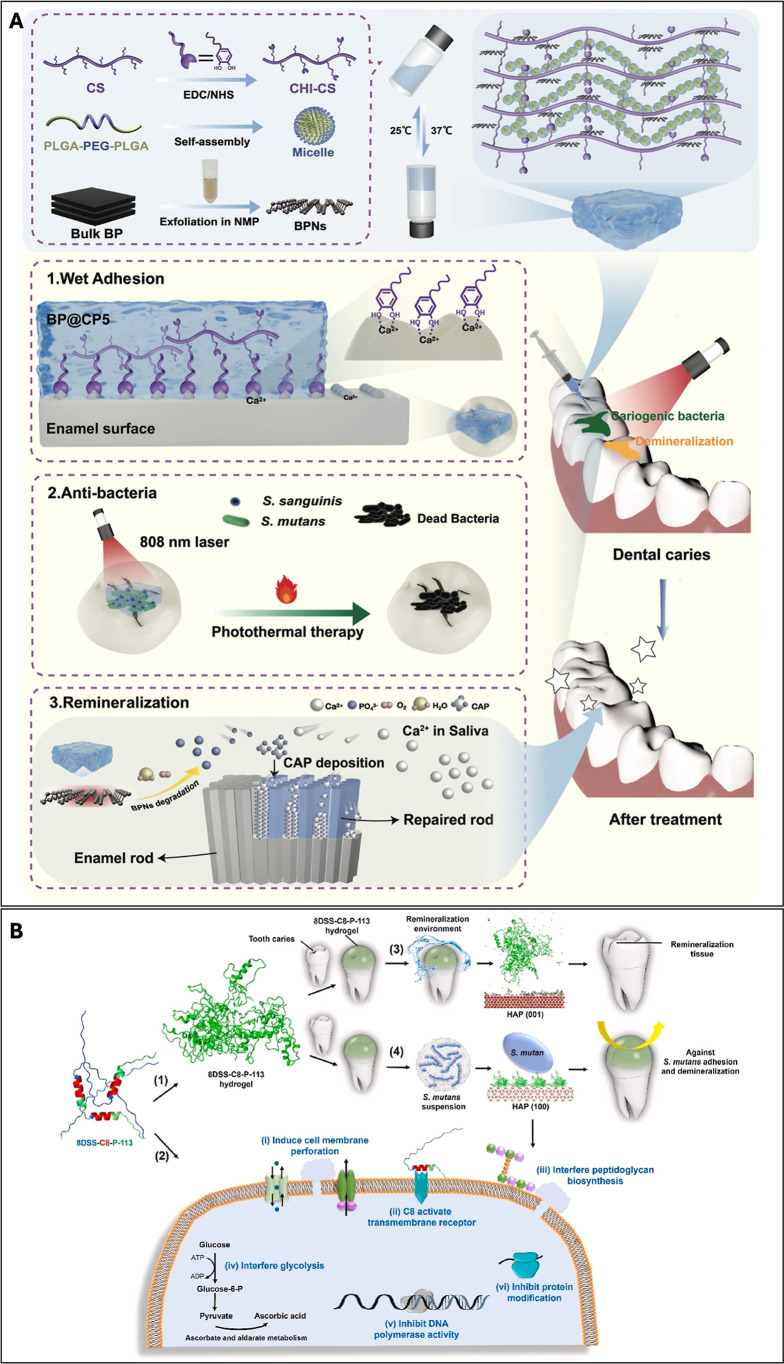
Two multifunctional remineralization system based on synthetic hydrogels. (A) Schematic illustration of BP@CP5 preparation and three main functions for caries management. Reproduced from ref 144. Available under a CC-BY license. Copyright ^©^ Ying Ran, Jiayi Shi, Yiqin Ding, Lujian Li, Dandan Lu, Youyun Zeng, Dongchao Qiu, Jie Yu, Xiaojun Cai, Yihuai Pan. (B) Schematic diagram of the functions of 8DSS-C8-P-113 and potential mechanisms of combating *S. mutans*. Reproduced with permission from ref 145. Copyright ^©^ 2025 ELSEVIER.

Peptide hydrogels, with artificially designed peptide sequences, have also displayed great potential in biomimetic mineralization. Zhou et al. designed and synthesized a novel multifunctional peptide, 8DSS-C8-P-113, which would self-assemble into a hydrogel at a specific concentration (16 μM/mL).^145^ The hydrogel state not only facilitated adherence to the tooth surface and the following functioning but also protected the underlying tooth surface from acid attack. 8DSS-C8-P-113 demonstrates specific antibiofilm activity, effectively eradicating *S. mutans* biofilms while preserving commensal oral flora, attributed to the broad-spectrum antimicrobial peptide (P-113) and the targeting peptide (C8). Moreover, owing to 8DSS, a domain rich in Asp–Ser–Ser repeats that enhance remineralization, the peptide hydrogel induces in situ remineralization of demineralized enamel with generation of a dense and oriented layer of HA crystals and substantial recovery of microhardness. Therefore, this multifunctional peptide, as shown in Figure 5(B), combining targeted antimicrobial activity, acid resistance, and guided biomimetic remineralization in an adherent hydrogel, represents a highly promising strategy for tooth structure repair and caries prevention.

## 6 Conclusion and Perspectives

This perspective explores the roles of polymers in biomimetic strategies for enamel and dentine remineralization. Polymers serve as versatile functional templates, effectively overcoming the limitations of conventional remineralizing agents by simulating key aspects of the natural biomineralization process. Previous reviews on materials for tooth remineralization have mentioned polymers; however, they did not focus exclusively on polymers and were generally organized according to polymer types. In contrast, this review classifies these polymers based on four distinct functional mechanisms of promoting remineralization: (1) simply accumulating more mineralization sources, usually Ca^2+^, to promote nucleation and further remineralization; (2) mimicking enamel matrix proteins to guide ordered enamel remineralization; (3) mimicking NCPs to facilitate intrafibrillar mineralization within the dentine collagen matrix; and (4) creating mineralization-favorable microenvironments, notably through hydrogel systems. Significant progress has been made using various polymers, including peptides, peptoids, polyphenols, polyelectrolytes, polysaccharides, etc., and numerous polymers remain to be explored for their potential roles in biomimetic mineralization. Meanwhile, the functions of these polymers often overlap: for instance, polymers that mimic enamel proteins or NCPs are primarily capable of attracting calcium ions, while those serving as matrices in hydrogels can simultaneously stabilize ACP and facilitate mineralization. This demonstrates that polymers promoting enamel and dentine mineralization exhibit diversity not only in type but also in function.

Over the years, polymer-involved strategies for enamel and dentine mineralization have evolved from single-function agents—capable only of promoting mineralization—into multifunctional polymeric systems. This is a prominent trend that these advanced systems not only promote remineralization but also address multiple challenges within the complex physiological and pathological conditions of the oral environment. Consequently, polymers that combine remineralization capabilities with antibacterial or antibiofilm activity, collagen protection, pH-responsiveness, or thermal-responsiveness have emerged as particularly promising platforms for practical tooth repair and effective caries management.

However, challenges persist in the advancement of enamel and dentin remineralization, involving polymers. Here, these challenges, alongside possible future directions, are discussed.

First, while the role of polymers in enamel and dentine remineralization is well established, recent studies have explored the use of removable polymers or small molecules to guide mineralization processes, particularly for enamel. For example, in two aforementioned studies, RNA and starch were hydrolyzed by RNase and α-amylase, respectively, to eliminate the influence of polymers on the formation of highly mineralized, organics-free mature enamel.^62,63^ Additionally, triethylamine (TEA), a volatile small molecule, was demonstrated to stabilize CPICs, enabling the formation of an amorphous precursor layer that could induce the epitaxial growth of enamel apatite.^146^ TEA was volatilized through ethanol evaporation, ensuring the removal of organics and the production of a purely inorganic structure. Small molecules such as citrate also promote dentine repair. Citrate adsorbs onto collagen and enhances biomimetic mineralization by modulating the wetting effect between ACP and collagen.^147^ These findings raise important considerations for future research: whether to incorporate polymers into mineralization systems, the specific roles polymers play at different stages of biomimetic mineralization, and whether small molecules or polymers yield superior mineralization outcomes. However, polymers still possess advantages over small molecules: (1) polymers can form complex structures (e.g., self-assemblies, hydrogel networks, dendrimers, etc.) that are unachievable with small molecules. Thus, polymer-based materials can exhibit multifunctionality or serve as versatile platforms, such as the rationally designed peptide 8DSS-C8-P-113, which integrates mineralization and *S. mutans*-specific antibacterial activity through distinct peptide sequences, or PAMAM dendrimers capable of delivering small-molecule drugs.^108,145^ (2) The oral cavity is a dynamic environment where saliva, enzymes, and pH fluctuate. Small molecules, more susceptible to removal and degradation,^148,149^ may be less stable than polymers in the oral environment. Taken together, the choice between polymers and small molecules should be objective dependent. Small molecules or removable polymers offer advantages when the goal is to achieve a highly pure, organic-free enamel structure or for localized, short-term application. While for mineralization of dentin, which inherently composes a higher proportion of organics compared to enamel, or for the construction of multifunctional materials, polymers retain significant advantages and promise.

Second, most current research on enamel and dentine mineralization has focused on creating enamel-like or dentine-like structures on the surface of demineralized slices. However, these structures still do not fully replicate the intricate architecture of natural enamel and dentine. Furthermore, these studies primarily rely on observations of crystal morphology, arrangement, and growth rates, but the lack of standardized evaluation criteria hampers rigorous comparisons of the efficacy among different polymers in biomimetic mineralization. Most of the polymer-based strategies remain at the preliminary research stage, lacking in vivo animal studies and clinical trials, and most investigations have been conducted under simplified conditions that fail to replicate the complexity and dynamic nature of the oral environment. These factors collectively indicate that, except for a few polymers that have been successfully translated into commercial products (e.g., CPP and P_11_-4), the majority of these mineralization approaches still have a considerable distance to cover before clinical translation can be realized. Therefore, future research should aim to advance our understanding of dental hard tissue remineralization by focusing on several key areas: (1) Continue to explore the underlying mechanisms in depth, identifying key factors involved in reconstructing hard tissues and providing insights into how to truly replicate enamel and dentin structures. (2) Establish an evaluation system conducive to clinical translation, assessing more clinically relevant metrics under conditions that better simulate the real complex oral environment (e.g., the recovery of mechanical properties and caries resistance). In addition, changes in crystal characteristics should be quantified, rather than relying solely on microscopic observation. (3) Consider the potential for clinical translation from the early stages of research design, developing patient-friendly delivery systems and administration methods, such as gels, films, and mouthwashes.

Third, our understanding of the roles played by polymers in biomineralization remains incomplete. The same polymer can serve multiple functions. For instance, chitosan may act as a pretreatment agent to promote mineralization, serve as an ACP stabilizer after carboxylation, or function as a raw material for hydrogels with antibacterial competence as well. Additionally, while the strategies discussed in this review are cell-free approaches, it is important to note that in cell-based tissue regeneration—such as enamel or dentine regeneration—polymers also play crucial roles, primarily as scaffolds, which are one of the three key elements of tissue engineering. Although some literature in the “Enamel proteins and mimics” section claims to achieve “enamel regeneration”, this does not refer to cellular enamel regeneration in the strict sense but rather to the regeneration of enamel-like structures, which is still remineralization.^150^ Therefore, the diverse roles of various polymers in enamel and dentine repair warrant further comprehensive exploration. Future efforts should focus not only on discovering new polymers but also on thoroughly investigating the multifunctional potential of existing polymers to maximize their applications in biomimetic mineralization and tissue regeneration.

## Data Availability

All data included in this study are available upon request from the corresponding author.

## Abbreviations

HA: hydroxyapatite
DOPA: 3,4-dihydroxy-l-phenylalanine
PDA: polydopamine
SEM: scanning electron microscopy
ACP: amorphous calcium phosphate
TA: tannic acid
WSLs: white spot lesions
PAA: poly­(acrylic acid)
SAP: salivary-acquired pellicle
BAG: bioactive glass
CaP: calcium phosphate
CPPs: casein phosphopeptides
ACFP: amorphous calcium fluoride phosphate
FHA: fluoridated hydroxyapatite
LRAP: leucine-rich amelogenin peptide
TRAP: tyrosine-rich amelogenin peptide
PTL: phase-transited lysozyme
PAMAM: polyamidoamine
PNCs: prenucleation clusters
CMC: carboxymethyl chitosan
ALN: alendronate
AuNPs: gold nanoparticles
Pchi: phosphorylated chitosan
CaP–PNCs: calcium phosphate prenucleation clusters
EGCG: epigallocatechin gallate
aMSNs: amine-functionalized mesoporous silica nanoparticles
PCBAA: poly­(carboxybetaine acrylamide)
NCPs: noncollagenous proteins
DSPP: dentine sialophosphoprotein
DMP-1: dentine matrix protein 1
BSP: bone sialoprotein
MEPE: matrix extracellular phosphoglycoprotein
SIBLINGs: Small Integrin-Binding Ligand N-linked Glycoproteins
DPP: dentine phosphoprotein
DSS: aspartate-serine-serine
PILP: polymer-induced liquid precursor
pAsp: poly­(aspartic acid)
RMGI: resin-modified glass ionomer
PCF: phosphate-containing fluid
PVPA: polyvinylphosphonic acid
PAH: poly­(allylamine hydrochloride)
STZ: silica/mesoporous titanium zirconium
NaF: sodium fluoride
PNG: PAMAM-NGV@galardin
MMPs: matrix metalloproteinases
PA: proanthocyanidin
ODAM: odontogenic ameloblast-associated protein
AMTN: amelotin
SBF: simulated body fluid
CS-AMEL: amelogenin-containing chitosan
PVP: poly­(vinylpyrrolidone)
TDM: treated dentine matrix
CHI-CS: catechol-modified chitosan
BPNs: phosphorus nanosheets
PPP: PLGA-PEG-PLGA
HPMC: hydroxypropylmethylcellulose
ELRs: elastin-like recombinamers
Tt: transition temperature
TEA: triethylamine
CPICs: calcium phosphate ion clusters
